# A prognostic model of drug tolerant persister-related genes in lung adenocarcinoma based on single cell and bulk RNA sequencing data

**DOI:** 10.1016/j.heliyon.2023.e20708

**Published:** 2023-10-05

**Authors:** Zhonghai Du, Tongtong Zhang, Yanke Lin, Guifen Dong, Aixiang Li, Zhiqiang Wang, Yongjie Zhang, Georgios Giamas, Justin Stebbing, Liping Zhu, Ling Peng

**Affiliations:** a Department of Medical Oncology, Weifang Hospital of Traditional Chinese Medicine, Weifang, Shandong Province, China; b Department of Medical Oncology, National Cancer Center/National Clinical Research Center for Cancer/Cancer Hospital & Shenzhen Hospital, Chinese Academy of Medical Sciences and Peking Union Medical College, Shenzhen, Guangdong Province, China; c Guangdong TCRCure Biopharma Technology Co., Ltd, Guangzhou, Guangdong Province, China; d Hospital Infection-Control Department, Shouguang Hospital of Traditional Chinese Medicine, Shouguang, Shandong Province, China; e Department of Medical Oncology, Shouguang Hospital of Traditional Chinese Medicine, Shouguang, Shandong Province, China; f Department of Urology, Shouguang Hospital of Traditional Chinese Medicine, Shouguang, Shandong Province, China; g Department of Biochemistry and Biomedicine, School of Life Sciences, University of Sussex, Brighton, United Kingdom; h Division of Cancer, Department of Surgery and Cancer, Imperial College London, London, United Kingdom; i Department of Biomedical Sciences, Anglia Ruskin University, Cambridge, United Kingdom; j Cancer Center, Department of Pulmonary and Critical Care Medicine, Zhejiang Provincial People's Hospital, Affiliated People's Hospital, Hangzhou Medical College, Hangzhou, Zhejiang, China

**Keywords:** Drug tolerant persister, Non-small cell lung cancer, Epidermal growth factor receptor, Tyrosine kinase inhibitor, Single-cell RNA sequencing, Bulk RNA sequencing

## Abstract

**Background:**

Acquired resistance to targeted drugs is a major challenge in cancer. The drug-tolerant state has been proposed to be an initial step towards acquisition of real drug-resistance. Drug tolerant persister (DTP) cells are purported to survive during treatment and stay dormant for several years. Single cell sequencing can provide a comprehensive landscape of gene expression in DTP cells, which can facilitate investigation of heterogeneity of a drug tolerant state and identification of new anticancer targets.

**Methods:**

The genetic profiling of DTPs was explored by integrating Gene Expression Omnibus (GEO) datasets, and a prognostic signature of DTP-related genes (DTPRGs) in lung adenocarcinoma of TCGA LUAD cohort was constructed. The scores of infiltrating immune cells were calculated and activity of immune-related pathways was evaluated by single-sample gene set enrichment analysis (ssGSEA). Functional enrichment analysis of the DTPRGs between low- and high-risk groups was performed. Immune cell subtypes and immune-related pathways were analyzed.

**Results:**

An 11-gene panel (*MT2A, UBE2S, CLTB, KRT7, IGFBP3, CTSH, NPC2, HMGA1, HNRNPAB, DTYMK, and IHNA*) was established. DTPRGs were mainly correlated with nuclear division, chromosome segregation, and cell cycle pathways. Infiltration of immune cells was lower in the high-risk group while the inflammation-promoting and MCH-class I response pathway had higher activity in the high-risk group. A nomogram was generated with prognostic accuracy, further validated using clinical outcomes following therapy with epidermal growth factor receptor (EGFR) tyrosine kinase inhibitors (TKIs).

**Discussion:**

A prognostic model of lung adenocarcinoma based on DTPRGs was constructed. Targeting DTP cells is a potential therapeutic approach to prevent a drug tolerant state.

## 1 Introduction

The outcome of anti-cancer therapy is inevitably perturbed by drug resistance. A small population of tumor cells evade cell death from cytotoxic and targeted therapy by entering a slow proliferation but reversible state, known as the "drug tolerant persister (DTP)" state [1]. Evidence suggest that DTP cells without *bona fide* resistance mechanisms display a reversible phenotype characterized by reduced drug sensitivity and decreased cell proliferation, that can maintain residual disease and may serve as a reservoir for resistant phenotypes [2]. Residual tumors rely on DTP cells to evade treatment and give rise to disease relapse. The persister cells comprise a reservoir from which drug-resistant cancers may arise. Drug holidays at the drug-tolerant state enable tolerant cells to reverse into parental cells and become re-sensitized to the drug they were previously exposed to Ref. [3].

Drug tolerant persisters have been reported in various cancer types, models and therapies [1]. A similar phenomenon was also observed in patients receiving immune checkpoint inhibitors (ICI), with a subpopulation of immunotherapy persister cells (IPCs) identified that could resist CD8^+^ T cell-mediated cell killing [4]. In non-small cell lung cancer (NSCLC), patients with NSCLC characterized by activating mutations in the epidermal growth factor receptor (EGFR) clearly benefit from EGFR–tyrosine kinase inhibitor (TKI) therapy. However, EGFR-TKIs lead to acquired resistance and eventual tumor relapse, thereby affecting the patient survival and highlighting the urgent need to investigate novel strategies for circumventing drug resistance [5]. DTP cells majorly contribute to the development of drug insensitivity state and irreversible resistance to EGFR TKIs [6,7]. For instance, osimertinib, is an irreversible third-generation EGFR TKI that is approved as first-line treatment for EGFR mutation-positive NSCLC [8]. It is of vital importance to explore the mechanisms of tumor repopulating cells induced by osimertinib exposure.

The genes involved in drug tolerance are diverse and complex, reflecting that the process of DTP is rather multifaceted and requires urgent elucidation. The bulk sequencing of tumor tissues is not enough to depict the temporal and spatial status of a cancer patient. Single cell sequencing has superior performance, with comprehensive analysis of different subgroups within the tumor. It also provides the molecular landscape of each subpopulation to explore diverse cancer targets. Moreover, single cell sequencing can be integrated with bulk RNA-seq data deposited in public datasets, which could be used to construct biomarkers for predicting the clinical outcomes of cancer patients.

In this study, we described the spectrum of changes associated with drug tolerance in EGFR TKI-tolerant cancers. The drug tolerant persister-related genes (DTPRGs) in osimertinib-tolerant cancer were first screened based on a single cell sequencing dataset and a bulk RNA sequencing dataset. Then, a prognostic DTPRGs risk score was developed by univariate and multivariate cox analysis and LASSO method. Furthermore, a nomogram combining DTPRGs risk score and clinical variables were constructed. Our findings demonstrated that these drug tolerant persister-related genes play crucial roles in the process of lung cancer development and could be potential targets for treatment of lung cancer patients.

## 2 Materials and methods

### 2.1 Data collection

Single-cell RNA sequencing (RNA-seq) and corresponding information of GSE150949 were retrieved from GEO database for exploring potential DTPRGs, which contained 8139 cycling cell samples from PC9 cells treated with osimertinib. GSE153183 dataset with paired parental and TKI-induced persister cells were also downloaded. Batch effect was corrected by sva function in two different datasets. Besides, the RNA-seq fragments per kilobase of exon per million fragments mapped reads (FPKM) from TCGA-LUAD cohort were downloaded. Gene expression datasets GSE68465, GSE72094, GSE68571, GSE41271 and GSE31210 were used as external validation sets consisting of 926 lung adenocarcinoma patients.

### 2.2 Data processing and DTPRGs screening

The type of lung cancer cycling PC9 cells was isolated from the mixed total cell samples in GSE150949. Then, the count matrix and clinical information were combined to generate the object by “Seurat” package in R software. The poor quality of cells and genes were filtered out, and only the genes with more than only 5 cells detected and the cells detected more than 1500 gene numbers were selected. A false discovery rate (FDR) < 0.05 was set. Dimensions with significant separation were screened out through principal component analysis (PCA). The t-distributed stochastic neighbor embedding (t-SNE) algorithm was employed to perform dimension reduction for the top 20 principal components (PCs) and obtain the major clusters. Marker genes in each cluster were accessed with log2 [fold change (FC)] ≥1 and FDR <0.05, and the top 10 % of marker genes were laid out in the heatmap. Clusters were annotated based on marker genes through 'SingleR' package. Pseudotime and trajectory analyses of PC9 cells were performed by ‘Monocle’ package. Then, intracellular differentially expressed genes with distinct differentiation states were designated as DTPRGs with |log2 (FC)| ≥1 and FDR <0.05. The DTPRGs were screened by "limma" package for each set. Then, the tumor mutation burden (TMB) per megabase of each sample was calculated in the exome content.

### 2.3 Co-expression network construction

The expression profiles of DTPRGs between DTP and control group were extracted to construct weighted gene co-expression network (WGCNA) using “WGCNA” package. First, scale independence and mean connectivity were calculated with candidate soft-thresholding powers (1–30). The first candidate power was selected as the proper power with degree of independence >0.8. Then, a co-expression network was constructed. Modules were identified with the parameters mergeCutHeight set to 0.3 and minModuleSize set to 30. Finally, the Pearson correlation coefficient and *p* value between each module were calculated.

### 2.4 Construction and validation of the DTPRGs prognostic model

Cox regression analysis was employed to evaluate the correlations between each gene and survival status in the TCGA-LUAD cohort. The cut-off *p* value was set as 0.2 The least absolute shrinkage and selection operator (LASSO) Cox regression model was used to narrow down the candidate genes. Ultimately, 11 genes and their coefficients were obtained, and the penalty parameter (λ) was decided by the minimum criteria. The risk score was calculated after centralization and standardization of TCGA-LUAD data, and the risk score formula was as follows:

$$The\;risk\;score=\sum_{i=1}^{n}{Coef}_{i}\times {Expr}_{i}$$

where Expr_*i*_ represents the expression level of gene *i* and coef_*i*_ represents the regression coefficient of gene *i* in the signature.

The TCGA-LUAD patients were divided into low- and high-risk groups according to the median risk score, and the OS time was compared between the two groups. PCA based on the 11-gene signature was performed by “stats” package. The “survival”, “survminer” and “timeROC” packages were employed to perform a 5-year receiver operating characteristic (ROC) curve analysis. For the external validation, lung adenocarcinoma patients from the GEO databases (GSE68465, GSE72094, GSE68571, GSE41271 and GSE31210) were employed. The risk score was calculated by the same formula used for the TCGA cohort. The patients in the GEO cohort were also divided into low- or high-risk groups, and these groups were then compared to validate the model.

### 2.5 Prognostic analysis of the risk score

The clinical information (age, gender, and stage) of patients in the TCGA LUAD cohort were extracted, and the age and stage data of patients in the GEO cohort were also obtained. These parameters were analyzed with risk score in regression model. Univariate and multivariable Cox regression models were used. Then, a nomogram was constructed with the above factors through “rms”, “nomogramEx”, and “regplot” packages. Next, ROC and calibration curves analysis were performed.

### 2.6 Functional enrichment of the DTPRGs

Patients in the TCGA-LUAD cohort were stratified into low- and high-risk groups according to the median risk score. DEGs between two groups were filtered with |log2FC| ≥1 and FDR <0.05. Based on these DEGs, Gene ontology (GO) and Kyoto Encyclopedia of Genes and Genomes (KEGG) analyses were performed via “clusterProfiler” package. The single-sample gene set enrichment analysis (ssGSEA) via “gsva” package.

### 2.7 Statistical analysis

For violin plots, the Wilcoxon rank sum test was performed. Kaplan-Meier method with a two-sided log-rank test was used compare the OS of patients between subgroups. Univariate and multivariate Cox regression models were employed to assess the independent prognostic value of the risk model. Mann-Whitney test was used to compare the immune cell infiltration and immune pathway activation. All statistical analyses were carried out in R (version 4.0.3) and Perl software (version January 6, 7601), and *p* < 0.05 was considered to be statistically significant. ★ p＜0.05，★★ p＜0.01，★★★ p＜0.001.

## 3 Results

### 3.1 Quality control and normalization of data--108 DTPRGs was identified in GEO database

**Fig. 1:**
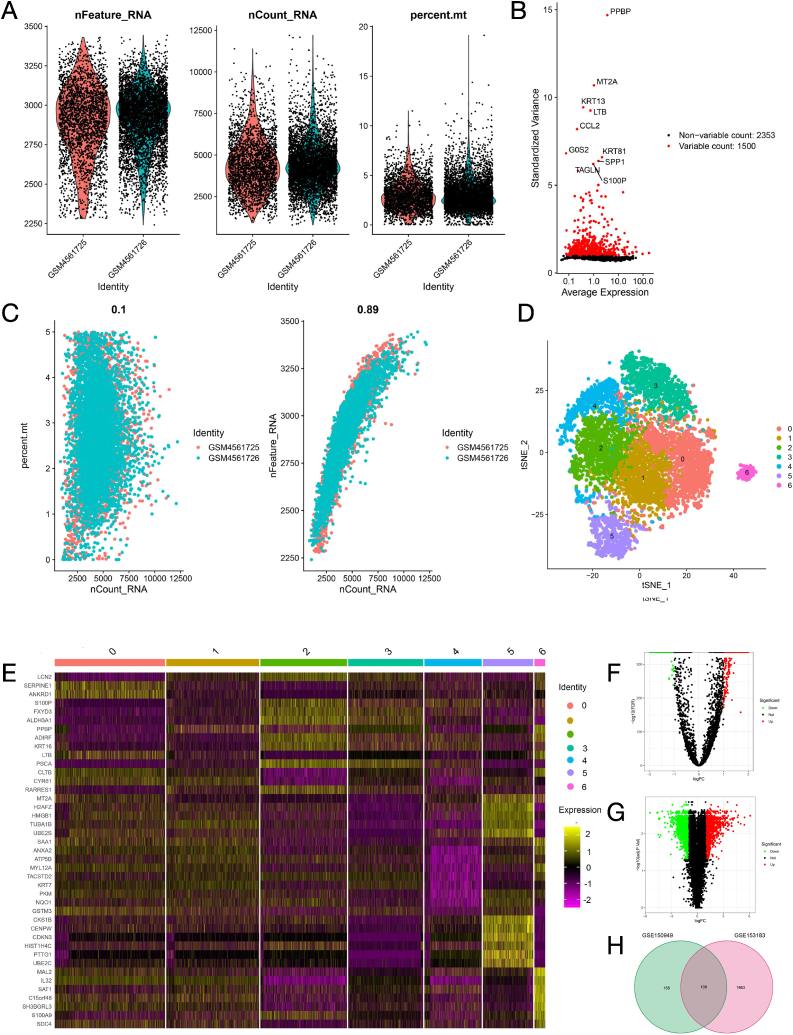
Quality control of single cell RNA-seq data and dimensionality reduction of differentially expressed DTPRGs. (A) Cells with poor quality were filtered out and the detected gene counts, mitochondrial gene sequences and sequencing depth were analyzed in sub-populations. (B) A subset of features that exhibit high cell-to-cell variation was calculated. Red dots indicate the 1500 variable genes. The top 10 gene names were labeled. nFeature_RNA indicates the numbers of genes detected in each cell. nCount_RNA indicates the total number of mRNA molecules detected in cells. Percent.mt indicates the ratio of mitochondrial gene expression to all cellular gene expression. (C) Correlation analysis between mitochondrial gene sequences and sequencing depth. nFeature_RNA indicates the numbers of genes detected in each cell. nCount_RNA indicates the total number of mRNA molecules detected in cells. Percent.mt indicates the ratio of mitochondrial gene expression to all cellular gene expression. (D) Cells in these 12 PCs were clustered into 7 types via t-SNE analysis. (E) The top 10 % marker genes of each cell cluster are displayed. The colors from purple to yellow denote low to high gene expression levels. Volcano plot of differentially expressed DTPRGs in GSE150949 dataset (F) and GSE153183 dataset (G). (H) Venn diagram of the overlapping DTPRGs in GSE153183 and GSE150949 datasets. (For interpretation of the references to color in this figure legend, the reader is referred to the Web version of this article.)

GSE153183 and GSE150949 datasets were analyzed. The study flow diagram is shown in Supplementary Fig. S1. Based on the selection criteria, 8139 cycling PC9 cell samples and 1500 high variable genes were identified in GSM4561725 and GSM4561726 samples derived from GSE150949 dataset. Fig. 1A shows the range of single cell RNA numbers and the RNA count of each cell, indicating a good quality control. Fig. 1B illustrates the 1500 high variable genes and the names of the top 10 genes of the cell samples. There was a significantly positive correlation between the gene numbers and sequencing depth with Pearson's r = 0.89 (Fig. 1C). According to the tSNE algorithm (Fig. 1D), 7143 PC9 cells are classified into 7 clusters, and the top 10 % of marker genes are displayed on the heat map, which represent low to high gene expression levels (Fig. 1E). Expression profile of GSE150949 displays 276 DTPRGs, in which 202 and 74 genes are upregulated and downregulated, respectively (Fig. 1F, Supplementary Table 1). Based on the cut-off values of |log2 (FC)| ≥1 and FDR <0.05, 1971 DTPRGs of persister cells were recognized in GSE153183 dataset, comprising 1007 upregulated and 964 down-regulated genes (Fig. 1G, Supplementary Table 2). We obtained 108 DTPRGs (36 downregulated and 72 upregulated genes) as the overlapping gene profile of persister by Venn plots (Fig. 1H).

WGCNA and identification of key modules--genes in key modules are mainly involved in nuclear division and chromosome segregation.

**Fig. 2:**
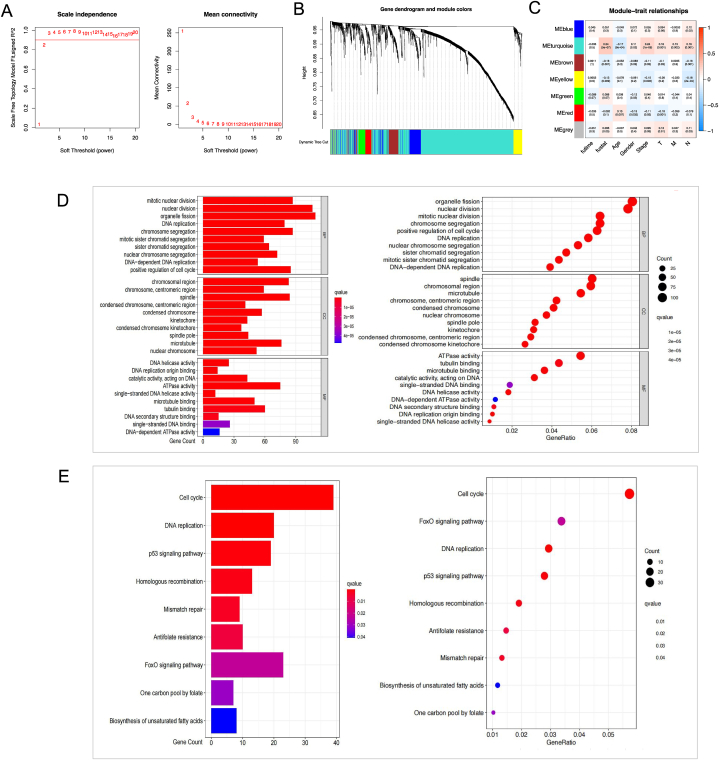
WGCNA of DTPRGs. (A–B) Based on WGCNA, 7 modules were accessed with a soft threshold = 2. (C) Correlation analysis between modules and clinicopathological data. (D–E) GO and KEGG were performed to reveal enriched functions and pathways of genes. futime: overall survival time. fustat: survival status. N: Nodes, describe regional lymph node involvement of the tumor; M: Metastasis, identify the presence of distant metastases of the primary tumor; T, tumor, identify the size and extension of the tumor. BP: biological process; CC: cellular component; MF: molecular function.

WGCNA on the TCGA-LUAD dataset was performed to find the key modules. After the intersection of DTPRGs in GSE153183 and GSE150949 sets, 2139 DTPRGs were enrolled in WGCNA. Seven modules were evaluated with soft threshold = 2 (Fig. 2A–B), of which 3 modules (e.g., blue, yellow module and turquoise module) were associated to overall survival (OS) of lung adenocarcinoma patients. The turquoise module was the most highly correlated with clinical traits from the heatmap of module-trait correlations (Fig. 2C). GO and KEGG analyses were performed to reveal biological functions of the genes within the turquoise module. Significant GO terms and KEGG pathways, are shown in Fig. 2D–E. This analysis indicates that genes within the turquoise module are mainly involved in nuclear division and chromosome segregation.

### 3.2 Identification of DTPRGs between normal and tumor tissues--hub genes were identified

**Fig. 3:**
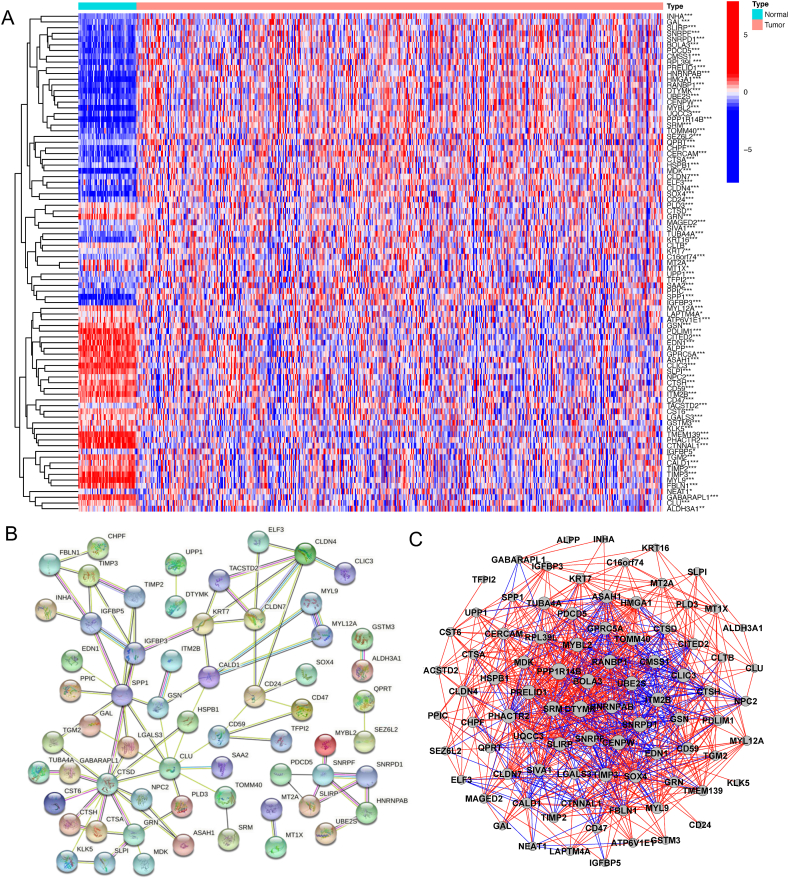
Expressions of the 87 DTPRGs and their interactions. (A) Heatmap of the DTPRGs between normal and tumor tissues. (B) PPI network displaying the interactions of the DTPRGs (interaction score = 0.4). (C) Correlation network of the DTPRGs. ★ p＜0.05，★★ p＜0.01，★★★ p＜0.001.

The expression levels of 108 DTPRGs were compared in TCGA data from 54 normal and 497 tumor tissues, and 87 differentially expressed genes (DEGs) were identified. Among them, 36 and 51 genes were downregulated and enriched in the tumor group, respectively (Table 1). The RNA levels of the genes are presented as heatmaps in Fig. 3A. A protein-protein interaction (PPI) analysis was conducted to explore the interactions of these DTPRGs (Fig. 3B). The minimum required interaction score was set at 0.4. *CTSD, HSPB1, KRT7, CLDN7, CD59, CLDN4, CLU, LGALS3, SPP1, CTSA, IGFBP3, IGFBP5, TIMP3, and TIMP2* were the top ranked genes and identified as hub genes. All the DEGs between normal and tumor tissues were among them. The correlation network of DEGs is presented in Fig. 3C.

**Table 1:** Genes downregulated and enriched in the tumor group.

Gene	Control Mean	Treatment Mean	logFC	p Value
SRM	15.88134749	35.424444	1.15741188	2.06E-24
TACSTD2	96.81181305	147.969664	0.61204643	0.00018221
GSTM3	7.239948051	6.12074745	−0.2422715	1.20E-06
ELF3	28.20461127	47.4591645	0.75075565	1.54E-10
LAPTM4A	188.0593729	179.491035	−0.0672764	0.02621899
BOLA3	2.28730478	4.64278049	1.02134046	5.46E-22
IGFBP5	42.33546807	71.2401195	0.7508231	0.03134925
TUBA4A	11.04153381	24.5976336	1.15557893	1.30E-16
CHPF	19.04167373	49.0135936	1.36402163	6.47E-23
INHA	0.086599977	6.96593477	6.32980651	6.79E-16
ALPP	2.603895178	1.0887751	−1.2579654	1.55E-22
DTYMK	5.187549542	13.7544015	1.40676825	2.77E-27
CMSS1	2.649281237	4.66380604	0.81590679	1.22E-15
CD47	43.88053136	33.2694578	−0.3993826	4.25E-10
RPL39L	2.823828246	15.6509476	2.47052576	6.22E-24
SPP1	9.264319714	288.483984	4.96066237	2.02E-30
PPIC	17.04960203	25.6050971	0.58669297	2.26E-12
CLTB	33.17878678	33.711363	0.02297382	0.02014324
PRELID1	15.6529859	24.3567422	0.6378833	1.69E-16
HNRNPAB	37.59750458	66.7657657	0.82847164	5.60E-26
EDN1	47.06018539	13.1677617	−1.8374969	2.09E-22
SOX4	12.76635058	44.1700264	1.79072152	1.60E-28
HMGA1	22.9883022	122.422038	2.41289146	2.54E-30
CD24	34.24429958	101.438084	1.56666365	1.47E-12
CENPW	3.143367932	9.63961325	1.61666412	4.35E-16
CITED2	92.15865288	51.5700207	−0.837587	6.84E-19
PHACTR2	9.295941729	3.82783629	−1.2800719	1.56E-27
IGFBP3	16.59999393	81.7615464	2.30023977	4.54E-21
UPP1	9.933659424	16.0850632	0.69532442	0.00014655
CLDN4	40.79847797	100.576167	1.30170124	2.07E-20
HSPB1	148.3443769	285.391138	0.94399029	5.99E-12
TFPI2	4.839896502	74.8138424	3.95025713	8.03E-06
CALD1	26.2254739	20.2917311	−0.3700769	4.92E-07
TMEM139	8.457735508	2.47120452	−1.775057	1.97E-27
MAGED2	54.91610881	70.5297018	0.36100154	2.79E-05
ASAH1	122.1930181	76.7542135	−0.670844	4.13E-19
CLU	75.81786915	65.3942888	−0.2133733	1.23E-08
CTNNAL1	29.24189932	12.1588143	−1.2660345	2.98E-26
GSN	64.51245847	43.6736822	−0.5628136	7.45E-12
CERCAM	3.406199169	10.2887203	1.59482886	2.26E-20
CLIC3	53.8552322	16.3428024	−1.7204311	9.36E-28
CTSD	619.5607203	509.802216	−0.281308	0.00308498
SAA2	1.099715268	3.70500319	1.75234474	2.10E-05
CD59	126.6893117	85.5787757	−0.5659699	4.07E-15
MDK	16.85298103	152.947007	3.18195616	4.49E-30
UQCC3	4.250509237	9.65515987	1.18366445	3.82E-23
PPP1R14B	12.9630129	59.8753495	2.2075611	9.08E-33
NEAT1	22.56929602	55.2825505	1.29246276	0.02505601
CST6	20.84141364	18.6593799	−0.1595521	6.62E-08
GAL	0.065680963	2.79633907	5.41192021	9.03E-18
PDLIM1	125.5037502	66.359371	−0.9193584	2.27E-26
GABARAPL1	33.37075983	18.381839	−0.8603034	4.37E-25
GPRC5A	173.218769	74.7220509	−1.2129893	6.74E-24
KRT7	112.13408	183.16169	0.70789297	0.00172326
SNRPF	7.534164441	13.7050399	0.86318711	7.23E-18
ITM2B	96.87619458	60.7401256	−0.6734923	2.30E-21
LGALS3	191.1799792	155.854448	−0.2947321	0.00039853
NPC2	268.1658356	231.48987	−0.2121764	3.79E-06
SLIRP	6.338896542	10.2414646	0.69211842	3.00E-17
SIVA1	9.471524169	12.2931572	0.37618697	0.00019354
CTSH	168.7091231	100.786091	−0.7432414	1.07E-16
QPRT	4.569890136	14.8933932	1.7044411	8.28E-06
SEZ6L2	7.154392085	25.8612587	1.8538914	5.39E-15
MT2A	163.5007936	105.753414	−0.6285934	0.0002088
MT1X	28.69912775	18.0674826	−0.6676114	0.01612824
C16orf74	0.608501698	2.04594128	1.74943155	3.91E-06
CLDN7	36.76869593	67.1194601	0.8682531	6.90E-15
ALDH3A1	5.632412695	21.5504834	1.93589528	0.00698081
KRT16	0.243273433	6.640135	4.77056189	1.51E-20
GRN	309.0352898	185.914957	−0.7331288	1.68E-17
TIMP2	86.81538085	59.1391771	−0.5538365	1.47E-12
MYL12A	126.9083739	101.80623	−0.3179614	1.24E-11
SNRPD1	5.186665661	9.83966914	0.92380243	2.66E-22
MYL9	171.8459269	57.9660916	−1.5678345	2.73E-32
TGM2	106.8153612	66.5277759	−0.6830904	8.43E-14
MYBL2	1.303018571	20.4471004	3.97196672	2.40E-31
SLPI	1398.956953	679.078094	−1.0427022	4.94E-22
CTSA	28.96906949	48.7664515	0.75137565	1.17E-15
PDCD5	10.82481786	22.1728672	1.03445259	1.13E-25
PLD3	95.29290424	93.3698963	−0.0294113	0.00085844
TOMM40	9.856123254	18.5469793	0.91209204	2.21E-19
KLK5	2.02510742	2.48360619	0.294438	2.24E-11
UBE2S	2.050522008	7.47530575	1.86614136	2.95E-22
ATP6V1E1	40.02451305	35.2233201	−0.184353	1.38E-06
RANBP1	6.734711627	14.1600264	1.07213588	1.00E-27
TIMP3	8.818162203	2.59914367	−1.7624416	3.00E-25
FBLN1	55.57887305	24.3550796	−1.1903139	7.84E-19

Abbreviations: LogFC, Log fold change.

### 3.3 Tumor classification based on the DTPRGs--DTPRSs was associated with tumor stage and survival

**Fig. 4:**
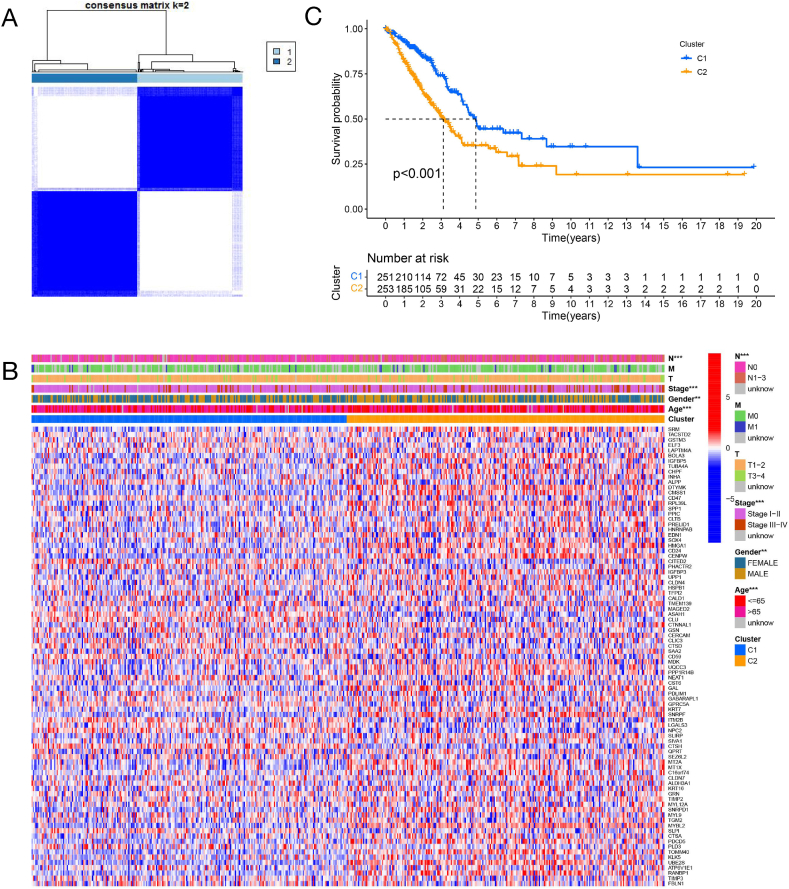
Tumor classification based on the DTPRGs. (A) 504 patients from TCGA LUAD cohort were grouped into two clusters according to the consensus clustering matrix (k = 2). (B) Heatmap and the clinicopathologic characters of the two clusters grouped by DTPRGs. (C) Kaplan-Meier OS curves of the two clusters. N: lymph node metastasis, describe regional lymph node involvement of the tumor; M: Metastasis, identify the presence of distant metastases of the primary tumor; T, tumor, identify the size and extension of the tumor. ★ p＜0.05，★★ p＜0.01，★★★ p＜0.001.

To investigate the correlations between the expression of the 87 DTPRGs and lung adenocarcinoma subtypes, a consensus clustering analysis was performed with all 504 patients in the TCGA LUAD cohort. With clustering variable (k) increased from 2 to 10, we found that when k = 2, the intragroup correlations are the highest and the intergroup correlations are low, indicating that the patients can be well divided into two clusters based on the 87 DTPRGs (Fig. 4A). The gene expression profile and the clinical parameters including lymph node metastasis (N), metastasis (M), tumor (T), stage, age, and gender are presented in a heatmap (Fig. 4B). We noticed that patients with early lung adenocarcinoma are associated with cluster 1, explaining why cluster 1 is associated with a better survival advantage (Fig. 4C). We also found that females and patients ≥65 are associated with cluster 1 and have a better overall survival.

### 3.4 A prognostic gene model in the TCGA cohort

**Fig. 5:**
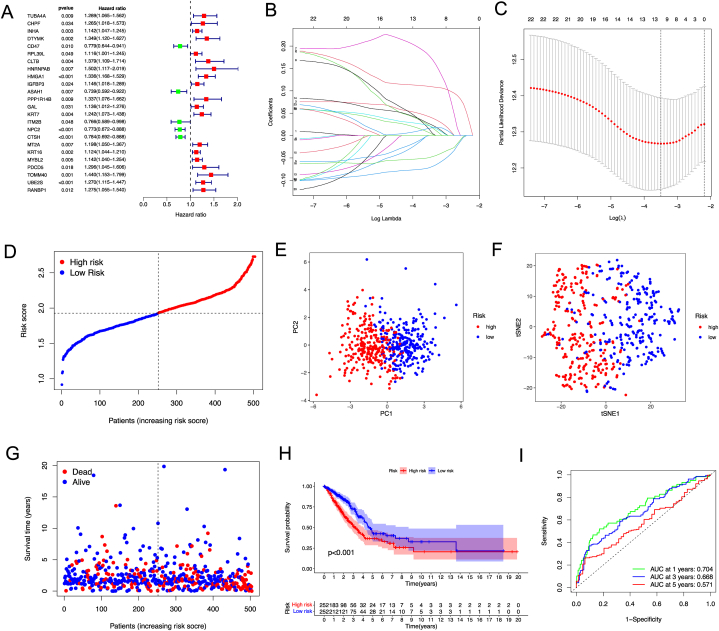
Construction of risk signature in the TCGA-LUAD cohort. (A) Univariate cox regression analysis of OS for 24 DTPRG with p < 0.2. (B) LASSO regression of the 11 OS-related genes. (C) Tuning parameter selection via cross-validation in the LASSO regression. (D) Distribution of patients based on the risk score. (E) PCA plot for PC9 based on the risk score. (F) t-SNE plot for OS based on the risk score. (G) Survival status of each patient (low-risk: left side of the dotted line; high-risk: right side of the dotted line). (H) Kaplan–Meier curves for the OS of patients in the high- and low-risk groups. (I) ROC curves demonstrated the predictive efficiency of the risk score.

A total of 504 samples of TCGA LUAD were matched to patients with complete survival information. Univariate Cox regression analysis was performed for initial screening of survival-related genes. The 24 genes (*TUBA4A, CHPF, INHA, DTYMK, CD47, RPL39L, CLTB, HNRNPAB, HMGA1, IGFBP3, ASAH1, PPP1R14B, GAL, KRT7, ITM2B, NPC2, CTSH, MT2A, KRT16, MYBL2, PDCD5, TOMM40, UBE2S, and RANBP1*) that met the criteria of *p* < 0.2 were submitted for subsequent analysis. Among them, except for *CD47, ASAH1, ITM2B, NPC2, and CTSH*, other genes were associated with increased risk (Fig. 5A). An 11-gene signature was constructed by LASSO Cox regression analysis according to the optimum λ value (Fig. 5B–C). The coeffient of 11-gene was listed in Table 2. The 504 patients were divided into low- and high-risk subgroups based on the median score (Fig. 5D). The PCA and t-SNE showed that patients are separated into two clusters (Fig. 5E–F). Patients in the high-risk group have a shorter survival time (Fig. 5G). A significant difference in OS time was observed between two groups (*p* < 0.001, Fig. 5H). Time-dependent ROC analysis indicated that the area under the ROC curve (AUC) is 0.704, 0.668, and 0.571 for 1-, 3-, and 5-year survival (Fig. 5I).

**Table 2:** The coeffient of 11 genes.

Gene	Coefficient
INHA	0.041831041
DTYMK	0.021908094
CLTB	0.177649624
HNRNPAB	0.013034096
HMGA1	0.109784999
IGFBP3	0.005979227
KRT7	0.026142311
NPC2	−0.048968943
CTSH	−0.071581479
MT2A	0.021425855
UBE2S	0.016344908

### 3.5 Prognostic value of the risk model

**Fig. 6:**
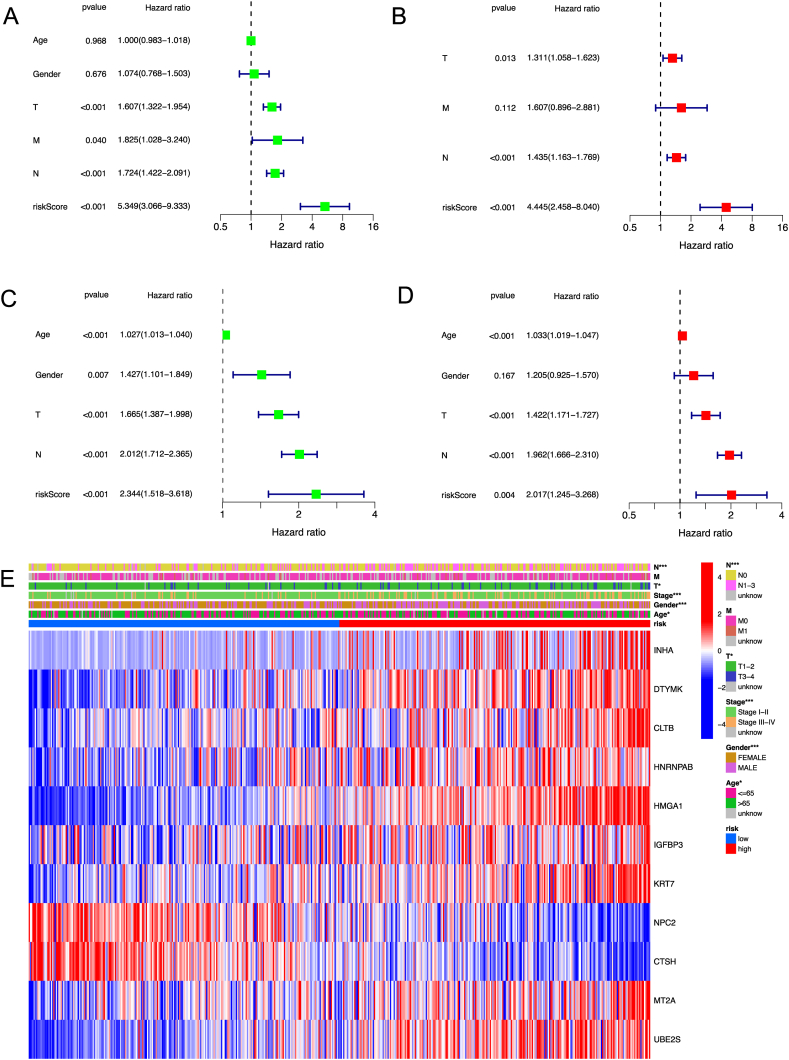
Univariate and multivariate Cox regression analyses for the risk score. Univariate analysis (A) and multivariate analysis (B) for the TCGA cohort. Univariate analysis (C) and multivariate analysis(D) for the GEO cohort. (E) Heatmap (blue and red indicate low and high expression, respectively) for the correlation of clinicopathologic features and the risk groups. ★ p＜0.05，★★ p＜0.01，★★★ p＜0.001. (For interpretation of the references to color in this figure legend, the reader is referred to the Web version of this article.)

The univariate Cox regression analysis indicated that the risk score is an independent factor predicting poor survival in both TCGA and GEO cohorts (HR = 5.349, 95 % CI: 3.066−9.333 for TCGA, and HR: 2.344, 95 % CI: 1.518−3.618 for GEO cohorts, Fig. 6A and C). The multivariate analysis also suggested that the risk score is a prognostic factor for patients with lung adenocarcinoma in both cohorts after adjusting for other confounding factors (HR = 4.445, 95 % CI: 2.458−8.040 for TCGA, and HR: 1.962, 95 % CI: 1.666−2.310 for GEO cohorts, Fig. 6B and D). The heatmap of clinical features for the TCGA cohort (Fig. 6E) found that gender, age, and the tumor stage are diversely distributed between the low- and high-risk subgroups.

### 3.6 Construction and validation of a nomogram

**Fig. 7:**
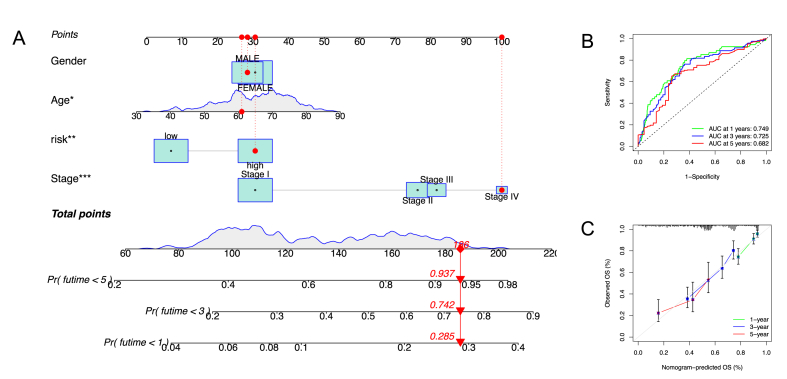
Construction and verification of the nomogram. (A) Nomogram constructed combining DTPRGs risk signature and clinical parameters. (B) The ROC curve and AUC of 1-, 3-, and 5-year of the nomogram for TCGA LUAD cohort. (C) The calibration plot of the nomogram for TCGA-LUAD cohort.

A nomogram was constructed combining the prognostic signature with clinical factors, such as age, gender and stage (Fig. 7A). The calibration curve of 45-degree line indicated the actual survival outcomes. The 1-, 3-, and 5-year OS demonstrated that the nomogram-predicted survival matched with the optimal prediction performance. The AUCs for the 1-, 3-, and 5-year OS were 0.749, 0.725, and 0.682, respectively (Fig. 7B). The calibration plot showed that the performance of the nomogram was an ideal model (Fig. 7C). These findings suggest that the nomogram combining the signature with clinical factors has a great prognostic accuracy.

### 3.7 External validation of the risk signature

**Fig. 8:**
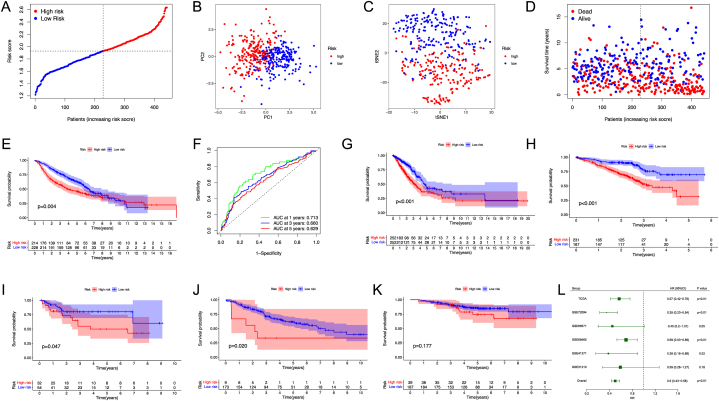
Validation of the risk model in the GEO cohort.

A total of 442 lung adenocarcinoma patients from GSE68465 were utilized as the validation set. Based on the median risk score of the TCGA LUAD cohort, 228 and 214 patients in the GEO cohort were classified into the low- and high-risk group, respectively (Fig. 8A). PCA and t-SNE showed well separation between the subgroups (Fig. 8B–C). Patients in the low-risk subgroup had longer survival times (Fig. 8D). Kaplan-Meier analysis demonstrated a significant difference in the survival rate between the groups (HR 0.69, 95 % CI: 0.53–0.89, log-rank test *p* = 0.004, Fig. 8E). ROC curve analysis showed that the model has good prognostic efficacy (AUC = 0.713, 0.660, and 0.629 for 1-, 3-, and 5-year survival, Fig. 8F).

The risk value for each patient in 5 independent GEO datasets were also calculated. Patients in different cohorts were separated into high- and low-risk groups. Kaplan-Meier analysis confirmed that patients in the high-risk group have a worse survival time, either in TCGA-LUAD cohort (Fig. 8G, HR 0.57, 95 % CI 0.42–0.76, log-rank test *p* < 0.01), GSE72094 (Fig. 8H, HR 0.35, 95 % CI 0.23–0.54, log-rank test *p* < 0.001), GSE68571 (Fig. 8I, HR 0.45, 95 % CI: 0.20–1.01, log-rank test *p* = 0.047), GSE41271 (Fig. 8J, HR 0.38, 95 % CI: 0.16–0.89, log-rank test *p* = 0.020), and GSE31210 (Fig. 8K, HR 0.59, 95 % CI: 0.28–1.27, log-rank test *p* = 0.177). The prognostic significance of DTPRGs-based signature in these public cohorts was determined by conducting a prognostic meta-analysis based on these 6 patient cohorts (n = 1838). The results confirmed that the DTPRGs based-signature is a risk factor for lung adenocarcinoma patients (combined HR = 0.59, 95 % CI: 0.28–1.27, meta-analysis *p* < 0.001) (Fig. 8L).

Functional analyses--the DTPRGs are mainly correlated with the nuclear division, chromosome segregation, and cell cycle pathways.

**Fig. 9:**
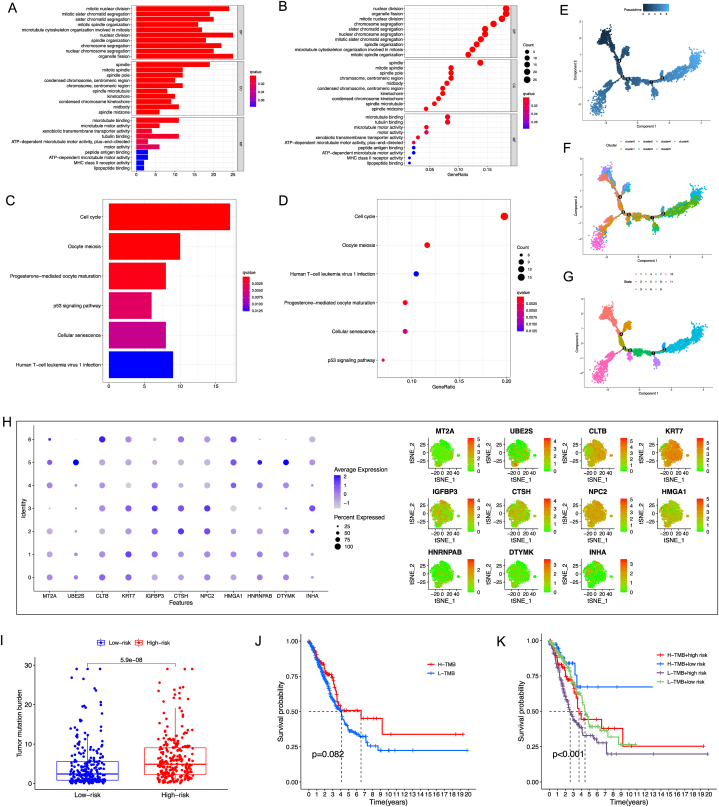
Functional analysis between risk groups in the TCGA-LUAD cohort.

In total, 139 DTPRGs between the low- and high-risk groups were identified in the TCGA cohort. Among them, 72 and 67 genes were upregulated and downregulated in the high-risk group, respectively (Table 3). GO enrichment and KEGG pathway analysis were performed based on these DTPRGs. The results indicated that the DTPRGs are mainly correlated with the nuclear division, chromosome segregation, and cell cycle pathways (Fig. 9A–D).

**Table 3:** DTPRGs between the low- and high-risk groups in the TCGA LUAD cohort.

Gene	Low Mean	High Mean	LogFC	p Value	FDR
SCGB1A1	7.262764919	6.12862567	−1.1341393	4.30E-05	0.00011407
KIF23	3.005492201	4.20211636	1.19662416	4.35E-34	1.88E-31
HLA-DQA1	6.970347064	5.83481057	−1.1355365	1.33E-08	6.67E-08
AQP4	5.099933102	3.87792628	−1.2220068	1.22E-16	2.39E-15
HJURP	4.269674918	5.42484574	1.15517082	2.94E-34	1.39E-31
TNNT1	3.512764535	4.55952586	1.04676132	9.00E-09	4.64E-08
PLA2G1B	4.579595234	3.37943476	−1.2001605	7.23E-19	1.99E-17
LMO3	7.111882669	5.87469175	−1.2371909	2.09E-19	6.28E-18
DPP4	6.675358928	5.54044583	−1.1349131	7.39E-15	1.06E-13
CDC45	2.973138627	4.52183069	1.54869207	1.96E-34	1.02E-31
RNASE1	10.24457164	8.6706471	−1.5739245	3.63E-30	6.50E-28
LRFN4	3.886588667	5.01400821	1.12741954	1.55E-22	7.44E-21
ABCA3	7.208863039	5.96484227	−1.2440208	2.07E-16	3.83E-15
CEP55	4.606227555	5.85614577	1.24991821	9.39E-34	3.75E-31
CDT1	3.890285276	4.9556403	1.06535503	3.97E-37	5.89E-34
MAD2L1	4.446346535	5.45845381	1.01210727	3.95E-28	5.07E-26
INHA	3.472008793	4.53339192	1.06138312	1.21E-13	1.47E-12
CYP4B1	6.934367592	4.89643041	−2.0379372	1.62E-23	9.07E-22
CACNA2D2	6.216074847	4.79900998	−1.4170649	2.17E-21	9.06E-20
MCM10	3.294219213	4.43771475	1.14349554	8.27E-31	1.69E-28
DMBT1	6.820071927	5.58021996	−1.239852	1.81E-11	1.48E-10
TLR2	6.551347122	5.45673556	−1.0946116	7.93E-28	9.81E-26
CXCL14	7.153297986	5.77291979	−1.3803782	5.71E-10	3.66E-09
BPIFA1	6.156384846	7.17283162	1.01644677	0.00204918	0.00392209
FCGBP	5.309548091	4.23915896	−1.0703891	1.40E-14	1.94E-13
FCER1A	4.582711464	3.44357066	−1.1391408	9.68E-22	4.24E-20
TNNC1	4.070679332	3.0630936	−1.0075857	2.16E-11	1.75E-10
TK1	6.085177364	7.0918202	1.00664284	1.39E-28	1.91E-26
CRYM	6.042238322	4.85130767	−1.1909307	3.83E-21	1.53E-19
ROS1	5.544383187	4.47887067	−1.0655125	2.52E-20	8.66E-19
TFF1	4.25065567	5.9588445	1.70818883	6.49E-12	5.78E-11
CPS1	3.767013702	5.14330927	1.37629557	2.25E-05	6.33E-05
ALOX15B	6.300463756	5.24778849	−1.0526753	1.80E-13	2.12E-12
CYP24A1	4.408690712	5.82293233	1.41424162	2.82E-14	3.75E-13
KRT16	2.450898192	3.7140932	1.26319501	8.38E-16	1.39E-14
ALPL	6.481853643	5.44158029	−1.0402734	2.11E-12	2.06E-11
SCGB2A1	3.604712049	2.58361728	−1.0210948	1.81E-08	8.89E-08
FOXM1	4.459827911	5.86405714	1.40422923	1.67E-35	1.27E-32
CDCA3	3.752814652	4.88403281	1.13121816	1.29E-35	1.12E-32
MELK	4.833533672	5.92670851	1.09317484	4.37E-27	4.46E-25
CCNA2	4.670033408	5.69432224	1.02428884	5.44E-32	1.38E-29
SLC7A5	6.358989238	7.38122727	1.02223803	1.03E-21	4.51E-20
CPA3	6.555687147	5.53831564	−1.0173715	1.61E-16	3.04E-15
LYPD3	3.820430317	5.0954309	1.27500058	2.23E-20	7.74E-19
TPX2	5.528220624	6.8863362	1.35811557	3.64E-35	2.10E-32
NFIX	4.785221293	3.74112935	−1.0440919	9.38E-28	1.15E-25
CDC20	5.019964973	6.60172177	1.58175679	6.69E-38	1.39E-34
MMP1	5.611119803	6.80474412	1.19362431	2.04E-11	1.66E-10
ST3GAL5	7.306211879	6.22270919	−1.0835027	6.43E-27	6.36E-25
SLC2A1	6.040625897	7.22301305	1.18238716	6.59E-33	2.21E-30
NCAPH	3.531704672	4.78334295	1.25163828	7.54E-31	1.57E-28
PAEP	3.823519019	4.9427103	1.11919128	2.49E-07	1.00E-06
CCNB1	5.495292368	6.68305554	1.18776318	3.28E-35	2.00E-32
KIFC1	4.292398204	5.63575818	1.34335997	1.15E-35	1.09E-32
SFTPC	8.631983745	6.56530614	−2.0666776	4.35E-12	3.96E-11
FSCN1	5.592113194	6.62812717	1.03601398	5.66E-21	2.24E-19
CDCA8	4.272832623	5.69888182	1.4260492	1.55E-32	4.75E-30
SELENBP1	8.522721698	7.23044016	−1.2922815	5.54E-26	4.64E-24
SLC22A3	5.161011121	3.75954707	−1.4014641	3.27E-21	1.33E-19
CD1C	4.548310256	3.29206737	−1.2562429	2.49E-20	8.59E-19
SLC47A1	4.350790186	3.13702757	−1.2137626	1.13E-22	5.47E-21
C4BPA	8.634426625	6.95133955	−1.6830871	2.78E-18	7.05E-17
APOH	4.914207301	3.81839708	−1.0958102	1.15E-14	1.61E-13
PIGR	8.177314489	6.05896794	−2.1183465	3.15E-29	4.75E-27
HLA-DRA	11.74301905	10.7032816	−1.0397374	1.31E-14	1.83E-13
CCNB2	5.052763021	6.20876048	1.15599746	2.81E-35	1.95E-32
AQP3	9.10427073	8.06485494	−1.0394158	1.05E-11	9.00E-11
KIF4A	4.324436041	5.38905141	1.06461537	2.66E-31	6.01E-29
FMO5	6.142954526	4.92546032	−1.2174942	7.26E-21	2.75E-19
UBE2C	6.380745198	7.73423591	1.35349071	6.64E-31	1.41E-28
FOSL1	3.678146376	5.15310739	1.47496102	9.80E-26	7.84E-24
RECQL4	3.228640916	4.24920861	1.0205677	1.59E-23	8.95E-22
PBK	4.176649648	5.25850451	1.08185486	1.99E-26	1.75E-24
UBE2S	5.38134744	6.54249657	1.16114913	3.28E-35	2.00E-32
TNS4	2.986633838	4.13586277	1.14922893	8.92E-15	1.27E-13
SCNN1B	6.243567534	5.1838151	−1.0597524	6.25E-18	1.48E-16
CYP2B7P	7.363986589	5.47687262	−1.887114	2.12E-26	1.85E-24
DUSP4	5.093540549	6.20641037	1.11286982	3.47E-14	4.56E-13
HOPX	8.650318147	7.30118737	−1.3491308	5.90E-16	1.00E-14
KIF11	4.275609625	5.28512795	1.00951833	3.80E-31	8.41E-29
IL37	3.31524359	2.03625906	−1.2789845	5.03E-16	8.63E-15
FGG	6.0671402	7.12430797	1.05716777	0.00018971	0.00044894
NUDT1	3.909678965	4.92261862	1.01293966	1.53E-21	6.54E-20
MYBL2	4.644573022	6.28451099	1.63993797	4.53E-36	5.23E-33
ABCC2	2.39242636	3.42483184	1.03240548	4.37E-05	0.00011577
BIRC5	4.98603523	6.13085544	1.14482021	2.65E-32	7.24E-30
ASPM	3.978712791	5.01713894	1.03842615	1.86E-24	1.22E-22
MFAP4	6.906590979	5.86212661	−1.0444644	6.21E-13	6.59E-12
PGC	7.870050268	6.09570042	−1.7743498	1.28E-11	1.08E-10
PRC1	5.043796434	6.07508601	1.03128958	5.56E-33	1.98E-30
NEK2	4.239907624	5.30963335	1.06972572	7.57E-29	1.06E-26
WIF1	5.130549296	3.60353841	−1.5270109	5.48E-14	6.95E-13
AURKB	4.160933004	5.30765945	1.14672645	1.71E-35	1.27E-32
RRM2	5.582700619	6.7990687	1.21636809	5.73E-33	1.98E-30
SFTPD	8.701811556	6.65645887	−2.0453527	3.12E-19	9.17E-18
SLC34A2	10.13516007	8.64791068	−1.4872494	7.61E-21	2.87E-19
HMGA1	7.252392931	8.59832856	1.34593563	3.44E-51	3.58E-47
CHRDL1	5.416413272	4.32814591	−1.0882674	2.46E-24	1.58E-22
ADH1B	5.46900291	3.93611976	−1.5328832	1.98E-25	1.47E-23
SCTR	4.870445581	3.32536287	−1.5450827	1.49E-27	1.76E-25
NKX2-1	8.079286818	6.6260199	−1.4532669	1.95E-19	5.86E-18
NDC80	3.289060412	4.55580368	1.26674326	3.38E-29	4.91E-27
CYBRD1	5.491985659	4.21015444	−1.2818312	3.30E-18	8.30E-17
CENPE	2.876467525	3.9702361	1.09376858	3.18E-27	3.37E-25
FGB	3.756735218	5.24433452	1.4875993	1.90E-08	9.30E-08
CD207	4.970622362	3.95388523	−1.0167371	1.33E-18	3.53E-17
CDA	3.975611203	5.2313084	1.2556972	1.80E-12	1.78E-11
PITX1	2.90194049	4.199679	1.29773851	7.06E-21	2.70E-19
GGTLC1	6.288214323	4.90442884	−1.3837855	8.61E-26	6.99E-24
DLGAP5	4.154122181	5.26332866	1.10920648	1.51E-30	2.97E-28
GPC4	7.062546602	6.05506404	−1.0074826	9.77E-24	5.77E-22
PARM1	7.460381159	6.41854129	−1.0418399	2.73E-20	9.29E-19
ARNTL2	4.196113092	5.19618068	1.00006759	3.02E-23	1.61E-21
TACC3	4.719046034	5.74268159	1.02363556	2.75E-32	7.32E-30
KRT6A	3.589668912	5.29089134	1.70122243	8.93E-19	2.44E-17
NDNF	7.081365095	5.842509	−1.2388561	8.05E-22	3.58E-20
SFTPB	11.94922208	10.0796892	−1.8695329	2.42E-17	5.25E-16
CTSH	9.711453582	8.24787623	−1.4635773	7.45E-41	2.58E-37
HLF	4.925814839	3.62782531	−1.2979895	1.76E-34	9.64E-32
GINS2	3.957261072	5.12215371	1.16489263	5.55E-24	3.35E-22
FGA	4.482942851	5.89288828	1.40994543	3.68E-06	1.19E-05
PI3	3.720862901	4.72473764	1.00387474	1.37E-13	1.64E-12
C7	6.585905794	4.97194626	−1.6139595	1.99E-22	9.31E-21
HSD17B6	5.751343414	4.36182226	−1.3895212	1.18E-23	6.83E-22
FOLR1	8.738060146	7.3447739	−1.3932862	8.30E-18	1.93E-16
NPC2	10.61008748	9.55257631	−1.0575112	2.59E-32	7.24E-30
AKR1B10	4.301663455	5.56285501	1.26119155	7.96E-06	2.43E-05
PTPN13	5.572084842	4.45528888	−1.116796	7.11E-23	3.58E-21
TMPRSS2	6.523716079	5.08390081	−1.4398153	4.65E-31	1.01E-28
CDKL2	3.663518334	2.48092067	−1.1825977	6.40E-37	8.31E-34
SERPINB5	2.630788997	4.24246987	1.61168087	2.77E-20	9.37E-19
PLK1	4.320317755	5.45304916	1.1327314	1.07E-41	5.58E-38
SERPIND1	4.407518498	3.26762002	−1.1398985	9.86E-11	7.25E-10
GJB3	3.810116354	4.92670552	1.11658916	4.46E-20	1.46E-18
KIF18B	4.31070324	5.32253063	1.01182739	1.44E-31	3.49E-29
S100P	7.346177349	8.7895048	1.44332745	6.59E-09	3.46E-08
ZNF750	4.389795839	3.25270432	−1.1370915	1.58E-17	3.53E-16
C1orf116	7.3375195	5.52414763	−1.8133719	1.34E-25	1.03E-23
TOP2A	5.826380385	6.86370119	1.03732081	1.64E-20	5.83E-19

Abbreviations: FDR, false discovery rate; LogFC, Log fold change.

Cell trajectory analysis, interrelation of the risk Score and TMB--the risk score is positively correlated with tumor malignancy.

According to the cell trajectories of PC9 cells, differential genes are scattered throughout the cell differentiation trajectory, with diversity and heterogeneity (Fig. 9E–G). The expression levels of 11 selected DTPRGs (*MT2A, UBE2S, CLTB, KRT7, IGFBP3, CTSH, NPC2, HMGA1, HNRNPAB, DTYMK, and IHNA*) in seven clusters are shown in Fig. 9H. Pseudotime and trajectory analysis indicated that MT2A and UBE2S increased in cluster 5 (belonging to state 1 and 9); CLTB in cluster 6 (belonging to state 7). We further found that there is a significant difference of TMB between high- and low-DTPRG associated prognostic model (DAPM) (Fig. 9I). However, there is no significant difference in OS between two groups (Fig. 9J). We then explored whether the combination of DAPM and TMB could be a better prognostic analysis. DAPM and TMB were integrated to stratify the samples into TMB^high^/DAPM^low^, TMB^low^/DAPM^low^, TMB^high^/DAPM^high^, and TMB^low^/DAPM^high^ groups. As shown in Fig. 9K, there are significant differences among all groups (Log-rank test,*p* < 0.001), and patients in the TMB^high^/DAPM^low^ group had the best OS. These results demonstrate that the risk score and tumor malignancy is positively correlated.

### 3.8 Immune activity between subgroups--the risk score is associated with immune activity

**Fig. 10:**
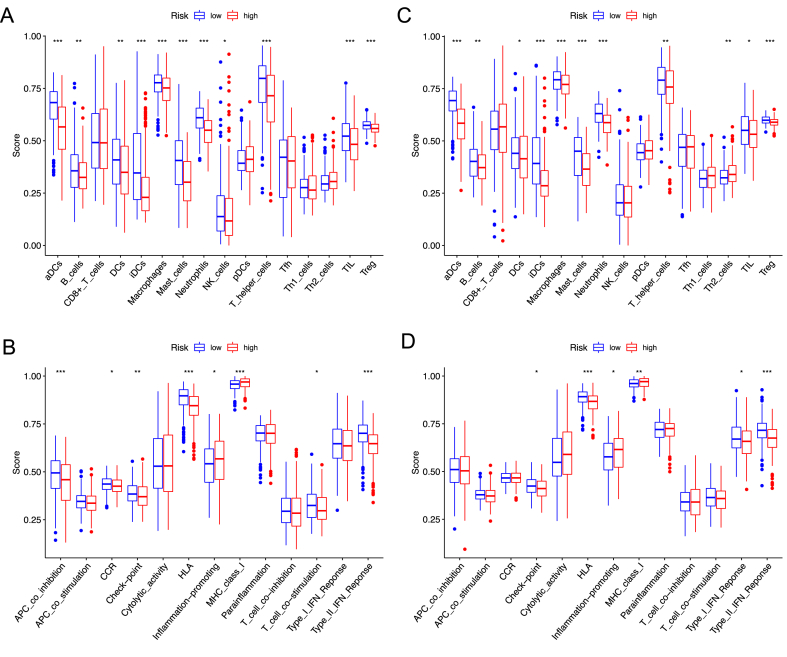
The ssGSEA scores for immune cells and immune pathways. (A, B) The enrichment scores of 16 types of immune cells and 13 immune-related pathways between low- and high-risk group in the TCGA-LUAD cohort. (C, D) the tumor immunity between low- and high-risk group in the GEO cohort.

The enrichment scores of 16 types of immune cells and the activity of 13 immune-related pathways between the low- and high-risk groups in both the TCGA and GEO cohorts were compared by ssGSEA. In the TCGA cohort (Fig. 10A), the high-risk subgroup had lower levels of immune cell infiltration, especially aDCs, B cell, iDCs, DCs, mast cells, macrophages, NK cells, neutrophils, tumor-infiltrating lymphocytes (TILs), T helper cells, and regulatory T (Treg) cells. The inflammation-promoting and MCH-class I response pathway showed higher activity in the high-risk group in the TCGA cohort (Fig. 10B). Similar conclusions were drawn with GEO cohort (Fig. 10C–D).

## 4 Discussion

In this study, pseudotime and trajectory analyses of single-cell RNA-seq data were carried out to screen DTPRGs. Bulk RNA sequencing data were also integrated with single cell RNA-seq data. WGCNA was used to explore potential genes associated with survival. Cox regression model was used to select genes and establish the prognostic model in TCGA LUAD cohort. A 11-gene signature of prognostic capability was constructed. External validation was performed using multiple GEO datasets of osimertinib-tolerant cell lines. The immune activity between subgroups was compared, and a nomogram of risk score combined with clinical characteristics was constructed and validated using external cohorts.

The results indicated that the biological processes in GO analysis related to DTP are nuclear division, organelle fission, chromosome segregation, mitotic nuclear division, positive regulation of cell cycle, and DNA replication, etc., which is consistent with previous studies demonstrating that the mitogenic and survival pathways are related to cells with slow-proliferating drug-tolerant phenotypes [9]. The mechanism of DTP state to EGFR TKI is diverse. The molecular mechanism behind the emergence of DTPs includes inactivation of AKT/Ets-1 signaling [10] and the expression of branched-chain amino acid aminotransferase (BCAT1) [11]. A metabolic shift to fatty acid oxidation and upregulation of antioxidant genes is associated with DTP state in multiple cancer types [12]. DTCs display markers associated with stem-cell like cancer cells [13]. A recent study using single-cell RNA sequencing confirmed the existence of diverse tumor cell subpopulations associated with EGFR TKI-tolerance, including epithelium development, epithelial-to-mesenchymal transition, drug metabolism vesicle mediated transport, and cholesterol homeostasis [8]. Mechanisms involving epigenetic modifications may also be responsible for DTP due to the reversible nature of drug tolerance [14], which is demonstrated by the fact that DTPs can be inhibited by treatment with chromatin-modifying drugs [13]. Studies of patient derived xenograft (PDX) models using DNA-barcodes for clonal tracking demonstrated that DTPs are more likely driven by a stochastic reprogramming process than a pe-existing clonal selection [15,16].

Altogether, 11 genes have been selected to construct a prognostic signature. *MT2A* gene encodes for metallothionein 2A protein. The levels of MT2A mRNA are considered as a marker of poor prognosis for lung cancer patients [17]. Additionally, inhibited MT2A expression resulted in cell death and apoptosis in prostate cancer cells [18]. *UBE2S* gene encodes for ubiquitin conjugating enzyme E2 S, which is a regulator of mitosis [19]. UBE2S contributes to lung cancer development by regulating canonical Wnt signaling [20]. UBE2S expression in lung cancer resulted in binding with IκBα to activate NF-κB signaling and cancer cell metastasis [21]. *Keratin 7 (KRT7)* is a member of the keratin gene family. Also known as cytokeratin-7 (CK7), KRT7 expression in lung cancer is associated with lymph node metastasis and T stage, which may serve as an independent factor for poor prognosis of lung cancer [22]. *IGFBP3* gene encodes Insulin Growth Factor Binding Protein 3, which is regulated by tumor suppressor p53. IGFBP-3 is responsible for transporting IGF-I and IGF-II in circulation, thus, prolonging the half-life of IGFs [23]. IGFBP3 could modulate cell growth and lung tumorigenesis through IGF1 signaling [24]. *CTSH* encodes for cathepsin H. Cathepsins are lysosomal enzymes belonging to the papain family. Cathepsins are involved in cancer progression and drug resistance [25]. *NPC2* encodes for NPC intracellular cholesterol transporter 2. The major function of NPC is to regulate cholesterol transport through lysosomal system [26]. NPC2 can be secreted by early-stage lung cancers and influence the tumor microenvironment [27]. *HMGA1* (High Mobility Group AT-Hook 1) has been related to lung cancer. Phosphoproteomic studies reveals HMGA1 as a drug-resistant target in NSCLC [28]. *HNRNPAB* encodes for heterogeneous nuclear ribonucleoprotein A/B (hnRNP AB), which belongs to the hnRNP families. Elevated expression of HNRNPAB was positively associated with more aggressive diseases and poorer survival rates in breast cancer [29]. *DTYMK* encodes deoxythymidylate kinase, which catalyzes biosynthesis of deoxythymidine triphosphate (dTTP). A previous study has shown that DTYMK is a predictive factor of poor prognosis in NSCLC [30]. *IHNA* (Inhibin Subunit Alpha) encodes TGF-β (transforming growth factor-beta) superfamily proteins. Elevated level of inhibin-α subunit is pro-tumorigenic and pro-metastatic in prostate cancer [31]. *CLTB* gene encodes clathrin light chain b. Upregulation of clathrin light chain b has been shown to couple with dynamin-1 in cancer cells, which leads to adaptive clathrin-mediated endocytosis and increases metastasis [32].

In our study, the high-risk subgroup had lower levels of immune cells infiltration and the inflammation-promoting and MCH-class I response pathway had higher activity in the high-risk group. Furthermore, combination of DAPM and TMB could be a powerful prognostic biomarker. The results demonstrated that our risk score has good capacity to separate lung adenocarcinoma patients of DTP into subgroups with different immune characteristics.

A variety of intracellular molecules are also DTP inducers in EGFR-mutated lung cancers, including Aurora kinase A, ERK, NF-kB, STAT3, and β-catenin [6]. The biologic features of DTP are not mutually exclusive and they often coexist within DTPs. They are regulated by upstream mechanisms such as transcriptional regulations, epigenetic modification, metabolic remodeling, and tumor microenvironment. Therefore, targeting a “master key” of these pathways may significantly contribute to the eradication of DTPs. DTPs lack proliferative ability, making them less harmful than cells with acquired resistance mechanisms. In clinical patients, DTP corresponds to minimal residual disease (MRD), which represents a transient phase between tumor response and progressive disease. Maintenance therapy such as chemotherapy may fit this concept. However, this strategy could not eradicate DTPs completely, and it requires long-term treatment, leading to many problems such as toxicity, patient compliance, and cost. As DTP cells can tolerate treatment and serve as the initial founders of acquired resistance, therefore, targeting DTPs would be essential for cancer control and prevention of acquired drug resistance.

The strength of our study is to incorporate single-cell RNA sequencing data with bulk RNA sequencing data to construct an 11-gene signature of prognostic value. Pseudotime and trajectory analysis has advantage to discover differentially expressed genes between lineages and to illuminate the underlying biological processes [33]. Combined with GO enrichments in WGCNA, the results demonstrated that persister cells maintained a dynamic persistent state, which is characterized by stress response and cell state transitions involving dynamic remodeling of chromatin architecture.

However, there are some limitations in our study. First, as different types of lung cancer need multiple clinical treatments, which will be heterogeneity of lung samples in the research and impact the accuracy of the results. Second, our data are based on publicly available database, the application of larger samples of clinical patients remain unanswered, which only preliminarily confirmed the reliability at the clinical level, but not thorough for establishing the practical guiding value in clinical practice. Third, the 11 genes selected to construct the signature has not been experimentally validated separately. Thus, to further establish the predictive significance of our prognostic signature, extensive prospective clinical research is necessary. In addition, this signature requires further validation in large prospective clinical trials.

In conclusion, we have developed a DTPRG signature in lung adenocarcinoma patients from single-cell and bulk RNA-seq data of GEO datasets. This demonstrates that there is a correlation between persister cells and patients’ survival. Our study provides insights into the mechanism and treatment for DTPs in lung adenocarcinoma patients.

Validation of DTPRGs-based signature in LUAD from different GEO datasets. Kaplan–Meier curves of OS in different GEO datasets. (A) Patients’ distribution in the GEO cohort based on the median risk score in the TCGA-LUAD cohort. (B) PCA plot for PC9. (C) t-SNE plot for PC9. (D) Survival status for each patient (low-risk: left side of the dotted line; high-risk: right side of the dotted line). (E) Kaplan-Meier curves for OS between low- and high-risk groups. (F) Time-dependent ROC curves for PC9. (G) TCGA-LUAD (n = 504); (H) GSE72094 (n = 398); (I) GSE68571 (n = 86); (J) GSE41271 (n = 182); (K) GSE31210 (n = 226). (L) A meta-analysis using the prognostic results of TCGA and GEO datasets.

Barplot graph (A) and Bubble graph (B) for GO enrichment. Barplot graph (C) and bubble graph (D) for KEGG pathways. (E) The direction of the cellular trajectory determined by unsupervised pseudo-time. (F) Cellular trajectory of PC9 cells subpopulations. The t-SNE plot indicated the distribution of single cells. (G) Trajectory analysis revealed 11 subsets of PC9 cells with distinct differentiation state. (H) Expression levels of DTPRGs in 7 clusters. (I) TMB between DAPM^high^ and DAPM^low^ groups. (J) Kaplan-Meier survival analysis based on the TMB in the TCGA-LUAD cohort. (K) Kaplan-Meier survival analysis for 4 groups by combining the TMB and the DAPM-based risk signature in the TCGA-LUAD cohort.

## Ethics approval and consent to participate

None applicable.

## Consent to publish

Not applicable.

## Availability of data and materials

Publicly available datasets were analyzed in this study.

## Funding

This study was partially supported by the 10.13039/501100004731Natural Science Foundation of Zhejiang Province, China (Grant number: LY19H160041) and a grant from the Administration of Traditional Chinese Medicine of Zhejiang Province, China (Grant number: 2022ZA021).

## Authors’ contributions

Zhonghai Du: Conceived and designed the experiments, Performed the experiments.

Tongtong Zhang: Wrote the paper.

Yanke Lin: Wrote the paper.

Guifen Dong: Analyzed and interpreted the data, Wrote the paper.

Aixiang Li: Analyzed and interpreted the data, Wrote the paper.

Zhiqiang Wang: Conceived and designed the experiments, Performed the experiments.

Yongjie Zhang: Analyzed and interpreted the data, Wrote the paper.

Georgios Giamas: Wrote the paper.

Justin Stebbing: Wrote the paper.

Liping Zhu: Wrote the paper.

Ling Peng: Conceived and designed the experiments, Performed the experiments.

## CRediT authorship contribution statement

**Zhonghai Du:** Conceptualization, Writing – original draft. **Tongtong Zhang:** Writing – review & editing. **Yanke Lin:** Writing – review & editing. **Guifen Dong:** Formal analysis, Writing – original draft. **Aixiang Li:** Formal analysis, Investigation. **Zhiqiang Wang:** Conceptualization, Formal analysis, Writing – original draft. **Yongjie Zhang:** Data curation, Writing – original draft. **Georgios Giamas:** Writing – review & editing. **Justin Stebbing:** Writing – review & editing. **Liping Zhu:** Writing – review & editing. **Ling Peng:** Conceptualization, Writing – review & editing.

## Declaration of competing interest

The authors declare the following financial interests/personal relationships which may be considered as potential competing interests:JS’ conflicts can be found at https://www.nature.com/onc/editors. GG is Editor in Chief in Cancer Gene Therapy and the Founder and Chief Scientific Advisor of Stingray Bio. None are relevant here. Other authors none declared.
