# Supplementary figures and images for: A prognostic model of drug tolerant persister-related genes in lung adenocarcinoma based on single cell and bulk RNA sequencing data
Source: Heliyon. 2023 Oct 5;9(11):e20708. doi: 10.1016/j.heliyon.2023.e20708 (PMC10618427; doi:10.1016/j.heliyon.2023.e20708)

# Supplementary Figure.


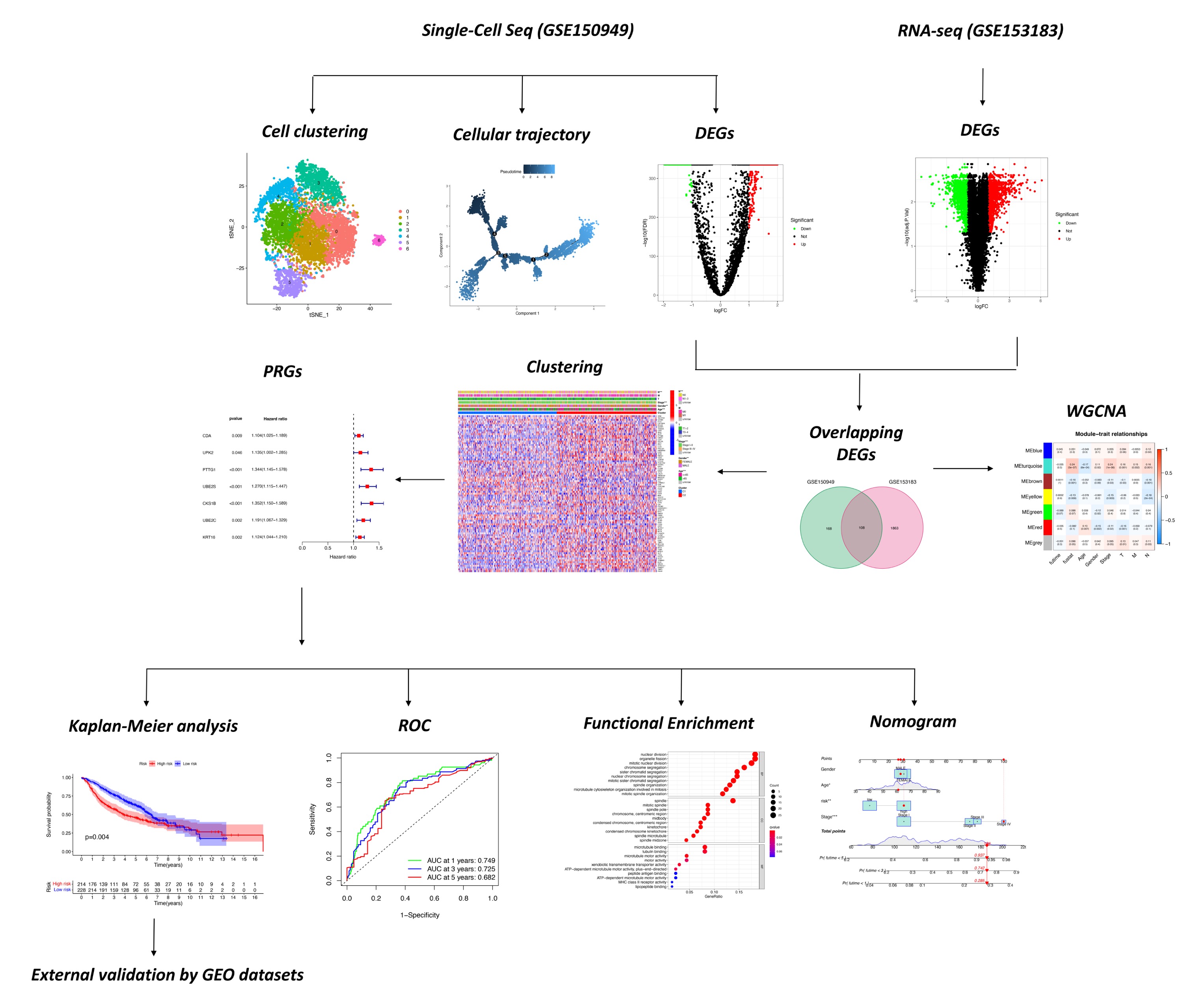


## Supplementary Figure S1. Study flow diagram.

Supplement: Multimedia component 1 [file mmc1.docx]
